# Molecular characterization of the transition from acute to chronic kidney injury following ischemia/reperfusion

**DOI:** 10.1172/jci.insight.94716

**Published:** 2017-09-21

**Authors:** Jing Liu, Sanjeev Kumar, Egor Dolzhenko, Gregory F. Alvarado, Jinjin Guo, Can Lu, Yibu Chen, Meng Li, Mark C. Dessing, Riana K. Parvez, Pietro E. Cippà, A. Michaela Krautzberger, Gohar Saribekyan, Andrew D. Smith, Andrew P. McMahon

**Affiliations:** 1 Department of Stem Cell Biology and Regenerative Medicine, Keck School of Medicine of the University of Southern California, Los Angeles, California, USA.; 2 Board of Governors Regenerative Medicine Institute, Cedars-Sinai Medical Center, Los Angeles, California, USA.; 3 Molecular and Computational Biology, Division of Biological Sciences, University of Southern California, Los Angeles, California, USA.; 4 Norris Medical Library, University of Southern California, Los Angeles, California.

**Keywords:** Nephrology, Molecular diagnosis, Mouse models

## Abstract

Though an acute kidney injury (AKI) episode is associated with an increased risk of chronic kidney disease (CKD), the mechanisms determining the transition from acute to irreversible chronic injury are not well understood. To extend our understanding of renal repair, and its limits, we performed a detailed molecular characterization of a murine ischemia/reperfusion injury (IRI) model for 12 months after injury. Together, the data comprising RNA-sequencing (RNA-seq) analysis at multiple time points, histological studies, and molecular and cellular characterization of targeted gene activity provide a comprehensive profile of injury, repair, and long-term maladaptive responses following IRI. Tubular atrophy, interstitial fibrosis, inflammation, and development of multiple renal cysts were major long-term outcomes of IRI. Progressive proximal tubular injury tracks with de novo activation of multiple Krt genes, including *Krt20*, a biomarker of renal tubule injury. RNA-seq analysis highlights a cascade of temporal-specific gene expression patterns related to tubular injury/repair, fibrosis, and innate and adaptive immunity. Intersection of these data with human kidney transplant expression profiles identified overlapping gene expression signatures correlating with different stages of the murine IRI response. The comprehensive characterization of incomplete recovery after ischemic AKI provides a valuable resource for determining the underlying pathophysiology of human CKD.

## Abstract 2

A comprehensive analysis of acute to chronic kidney injury transition in a mouse model with long-term survival after ischemia/reperfusion.

## Introduction

Acute kidney injury (AKI), an abrupt loss of kidney function, is associated with high morbidity and mortality (1–5). Recent clinical studies on the expanding populations of AKI survivors have linked AKI to an increased risk for progressive chronic kidney diseases (CKD) (2, 6–13). Despite extensive investigation of AKI in experimental models, the clinical outcome after AKI has not improved substantially in the last decade (3, 14, 15). The underlying mechanisms of AKI-to-CKD transition remain enigmatic.

Experimental animal models of renal ischemia/reperfusion injury (IRI) have been widely used to investigate the pathogenesis of ischemic AKI (16–18). Rodent injury models in particular enable powerful genetic, cellular, and molecular strategies to be applied to the analysis of injury outcome (19–23). Through these studies, key biomarkers have been identified that translate to renal injury responses in the human kidney (19, 21). However, there are reasonable questions as to how well rodent IRI models mimic human AKI, particularly with regard to species difference in immune system action, given the significance of immune responses to human renal pathophysiology (24–26). In consideration of the major challenges to developing an in-depth understanding of AKI in a heterogeneous patient population, rodent IRI models remain a valuable tool for mechanistic analysis, with the expectation that some, but not all, of the renal pathophysiological consequences will have human parallels.

Previous studies have applied genome-wide transcriptional analysis to AKI models in rodents, focusing predominantly on the early injury and reparative phase of the response (27, 28). In these models, persistent renal parenchymal injury responses are observed in conjunction with distinct chronic immune and inflammatory responses (19, 29–34). Many of these studies initiated IRI through renal pedicle clamping and release, mimicking conventional procedures in kidney surgery, for example, in removal of renal tumors, and kidney trauma associated with kidney transplant (29–32). Unilateral clamping and ureter obstruction performed in several studies (19, 33, 34) preclude the evaluation of renal dysfunction in relation to long-term survival, given the presence of a normal functioning kidney.

In this study, we developed a severe bilateral IRI recovery model on a genetically consistent background to explore the transition from acute to chronic injury in the mouse kidney. The molecular and cellular responses to a single IRI event were examined at 10 time points, from 2 hours to 12 months after injury. Analysis of comprehensive RNA-sequencing (RNA-seq) data sets identified stage-specific cohorts of gene expression correlating with distinct phases of the renal response, identifying cell type–specific responses from the whole-kidney data sets. Together, these studies provide insights into early and long-term responses to kidney injury and a resource for the clinical and research community. Further, individual genes highlighting distinct phases of the renal response in the mouse are potential diagnostic markers for exploring temporal progression to renal injury in humans.

## Results

### A murine model of AKI with progressive renal pathology.

**Figure 1:**
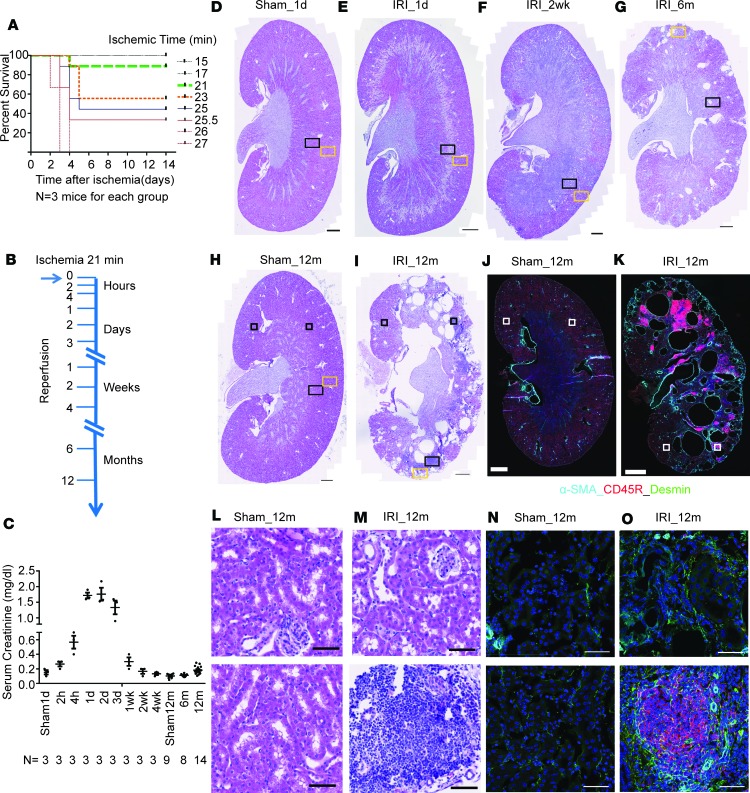
Incomplete recovery after 21 minutes of bilateral renal IRI. (**A**) Kaplan-Meier survival curves for mice with different periods of renal pedicle clamping (ischemia time). Mean is presented. (**B**) Sample collection time points. (**C**) Serum creatinine (mg/dl) after sham and IRI. Mean ± SEM is presented. (**D–I**) H&E staining on sagittal sections of the sham and IRI kidneys. Zoomed views for rectangular regions in **D–I** are shown in Supplemental Figure 1, G–L. (**J** and **K**) Immunostaining on sagittal sections of the kidneys 12 months after sham and IRI. α-SMA (cyan), CD45R (red), and desmin (green). Zoomed views for the top black squares in **H** and **I** are in **L** and **M**, with the left square corresponding to the top and right square to the bottom in **L** and **M**. Zoomed views for the white squares in **J** and **K** are in **N** and **O**, with the left square corresponding to the top and right square to the bottom in **N** and **O**. Scale bar: 1 mm (**D–K**); 50 μm (**L–O**).

To extend our understanding of the molecular mechanisms of disease progression after AKI, we set up a long-term survival renal IRI model in C57BL/6NCrl strain mice (Charles River). As our goal was to examine a survivable but severe injury, we performed an initial pilot study utilizing 9- to 11-week-old (25–28 g) male C57BL/6NCrl mice to identify the maximal ischemia time required to achieve a severe but survivable ischemic injury following bilateral IRI. Forty-five mice were subjected to varying periods of renal pedicle clamping. Survival analysis revealed 21 minutes as an ischemic time associated with a marked AKI response at 24 hours as well as a good survival rate of 14 days for over 90% of operated mice (Figure 1A). We adopted a 21-minute warm IRI throughout this study.

To obtain a comprehensive injury biobank, IRI was performed as above, and cohorts of injured mice were examined at 10 different time points following IRI: 2 and 4 hours; 1, 2, and 3 days; 1, 2, and 4 weeks; and 6 and 12 months (Figure 1B). Prior to sacrifice, blood and urine were collected for analysis of serum creatinine and proteinuria. Kidneys were collected for RNA extraction and RNA-seq analysis, standard histology, and in situ analysis of gene expression and key protein markers of specific cell types and injury responses.

As expected, bilateral renal injury resulted in markedly elevated levels of serum creatinine, peaking at day 2 after IRI (mean 1.76 mg/dl) and returning to baseline by 2 weeks after IRI (Figure 1C). Examination of mice at 12 months showed significantly elevated levels of serum creatinine and blood urea nitrogen (BUN) (Supplemental Figure 1, A and B; supplemental material available online with this article; https://doi.org/10.1172/jci.insight.94716DS1) and proteinuria in some survivors, relative to a surgical control group that did not undergo renal clamping (Supplemental Figure 1C).

Histological analysis of kidney sections at day 1 after IRI revealed significant tubular damage in the outer medullary region, as evidenced by cell flattening, sloughing of tubular epithelium, and denudation of tubular basement membrane and luminal casts zone, which were not seen in sham control (Figure 1, D and E, and Supplemental Figure 1H). Trichrome staining revealed that fibrosis was restricted to the outer medullary region adjacent to damaged S3 segments (Supplemental Figure 2, B and G). Two weeks after IRI, injured tubules showed squamous morphology with minimal cytoplasm and luminal casts were observed in medullary tubules (Figure 1F and Supplemental Figure 1I). A patchy cell infiltrate was evident in both cortical and medullary regions (Figure 1F and Supplemental Figure 1I), and trichrome staining highlighted collagen fiber deposition in the inner and outer medullary regions (Supplemental Figure 2H). By 6 months after IRI, patchy infiltrates of leukocytes, cortical fibrosis, and a few small cysts were seen (Figure 1G, Supplemental Figure 1K, and Supplemental Figure 2, D and I). By 12 months after IRI, kidneys exhibited larger focal cortical and medullary cysts and a marked dilation of the renal pelvis with severe chronic interstitial nephritis (Figure 1, H, I, L, and M) as well as prominent scarring (Supplemental Figure 2, E and J). Immune infiltration was observed over two-thirds of the kidney parenchyma (Figure 1, H, I, L, and M). The few remaining functional glomeruli were restricted to the anterior and posterior poles of the kidney (Figure 1, I and M). Fibrosis was confirmed by detection of α–smooth muscle actin (α-SMA) and desmin production within interstitial cells, and examination of CD45R highlighted a marked B cell infiltrate (Figure 1, J, K, N, and O). The profound, patchy tubular atrophy and numerous cortical cysts observed in fibrotic kidneys 12 months after injury are reminiscent of acquired cystic kidney disease, a feature of end-stage kidney disease in humans (35). Further, the highly organized CD45R^+^ B cell aggregates mirror human renal inflammation (36) and lupus nephritis (37), with features of tertiary lymphoid organs previously identified in kidney allografts (38), aged atherosclerosis-prone hyperlipidemic ApoE^–/–^ mice (39), and in aged, but not young, mice 45 days after AKI (40). In conclusion, a single acute injury invoked a progressive alteration of renal tissue, leading to histological findings normally associated with patients exhibiting advanced CKD, consistent with the goal of modeling an AKI-to-CKD transition.

### Cellular characterization of continuous proximal tubular injury, interstitial fibrosis, and immune cell infiltrates.

Recent studies have noted a change in cytokeratin expression (*Krt7/8/18/19*) in conjunction with renal tubule injury (41, 42). In particular, de novo expression of Krt18, normally restricted to collecting ducts and parietal epithelial cells of the Bowman capsule, was reported in the proximal and distal tubules of injured nephrons following 5 days of unilateral ureter obstruction (41). Consistent with these studies, we observed induction of these cytokeratin family members through translational profiling of AKI within Six2-derived nephron structures 4–24 hours after IRI, the microarray data of which was deposited at NCBI GEO (accession GSE52004) (28) (Supplemental Figure 3A). In addition, we observed a marked change in expression of *Krt20* (>50 fold; Supplemental Figure 3A), which had not previously been associated with either normal or injured kidneys.

**Figure 2:**
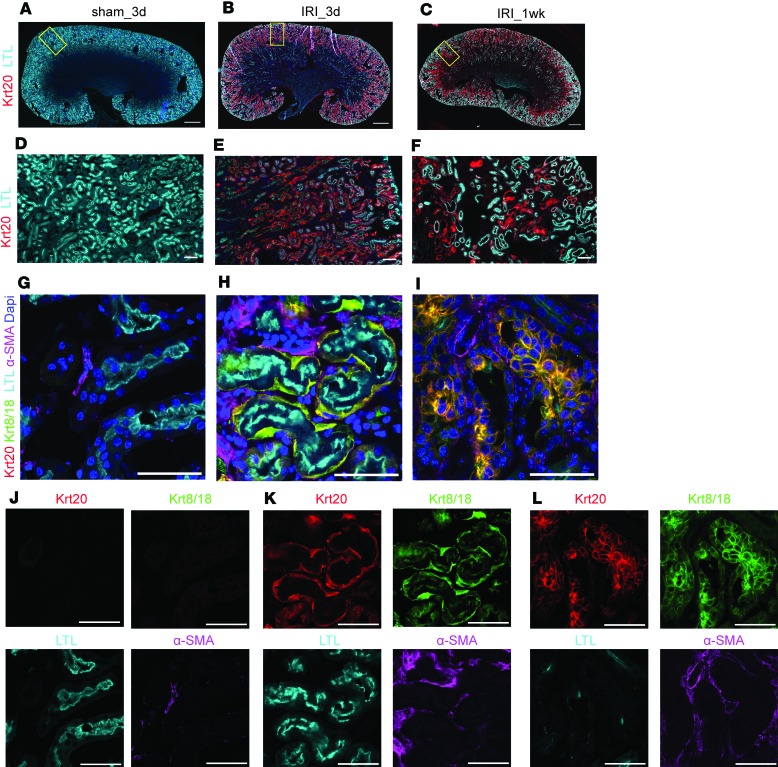
Continuous expression of Krt20/8/18 in injured proximal tubules. (**A–C**) Immunostaining on sagittal sections of kidneys 3 days and 1 week after sham and IRI. Krt20 (red) and LTL (cyan). (**D–F**) Zoomed views for the rectangular regions in **A–C**. (**G–I**) Confocal immunofluorescence of Krt20 (red), Krt8/18 (green), LTL (cyan), and α-SMA (magenta), showing the outer stripe of outer medullary regions on the sagittal sections of kidneys. (**J–L**) Split channel images for **G–I**. Scale bar: 1 mm (**A–C**); 50 μm (**D–L**).

We examined the distribution of keratins to assess renal tubule injury within the IRI biobank. In uninjured and sham surgery control kidneys, no Krt20 was detected in any renal tubule segment (Figure 2, A, D, G, and J). In contrast, by day 3 after IRI, Krt20 was readily detected in proximal tubules highlighted specifically through binding of FITC-conjugated Lotus tetragonolobus lectin (LTL), though LTL binding to proximal tubule was markedly reduced when compared with surgical (sham) control kidneys (Figure 2, B and E). At the junction of the cortex and outer medullary region, the majority of a sparse thin layer of elongated cells lining the LTL^+^ segment of the proximal tubule were colabeled by antibodies for Krt20, Krt8/18, and the kidney injury marker Havcr1 (also known as Kim1) (Figure 2, H and K, and Supplemental Figure 3, F, G, I, and J).

By 1 week after IRI, cuboidal Krt20^+^; Krt8/18^+^; LTL^–^ intratubular cells defined a subset of proximal tubules that had failed to repair highlighted with Havcr1^+^ (Figure 2, C, F, I, and L, and Supplemental Figure 3, H and K). Here, the tubular epithelium was surrounded by α-SMA–expressing interstitial myofibroblasts (Figure 2, I and L). By 4 weeks, extensive contiguous cortical tubular segments were Krt20^+^; Havcr1^+^; LTL^–^, indicative of atrophied cortical proximal tubules (Supplemental Figure 3, M and O); strong α-SMA accumulated around the former S3 segment of the proximal tubule within the outer stripe of the medullary region, indicative of interstitial fibrosis (Supplemental Figure 3, C and E).

In parallel to tubular damage and interstitial fibrosis, blood vessels and capillaries were affected, as evident from a loss of PECAM1^+^ (also known as CD31) vascular cells (Supplemental Figure 4, A–F): a 20%–40% reduction in vessel density was measured in the cortex and outer medulla after IRI (Supplemental Figure 4G). Innate and adaptive immune responses were suggested by the infiltration of F4/80^+^ macrophages (Supplemental Figure 5D) and CD4^+^ T cells in the medullary zone 2 weeks after IRI (Supplemental Figure 5F). Quantification of the CD45^+^ cohort of immune cells confirmed an increase of the F4/80^+^ macrophage population, which was followed by elevated levels of CD4^+^ T cells 4 weeks after IRI. At 1 year after IRI, the majority of lymphocytes in the CD45^+^ aggregates comprised both CD3^+^/CD4^+^ T cells and CD45R^+^ B cells(Supplemental Figure 5G). Together, these findings are consistent with an immunologically active fibrotic kidney, with extensive unresolved tubular injury that is nevertheless able to maintain basic renal activity for up to 12 months after IRI (Figure 1C).

### RNA-seq analysis identified temporal-specific gene regulation.

**Figure 3:**
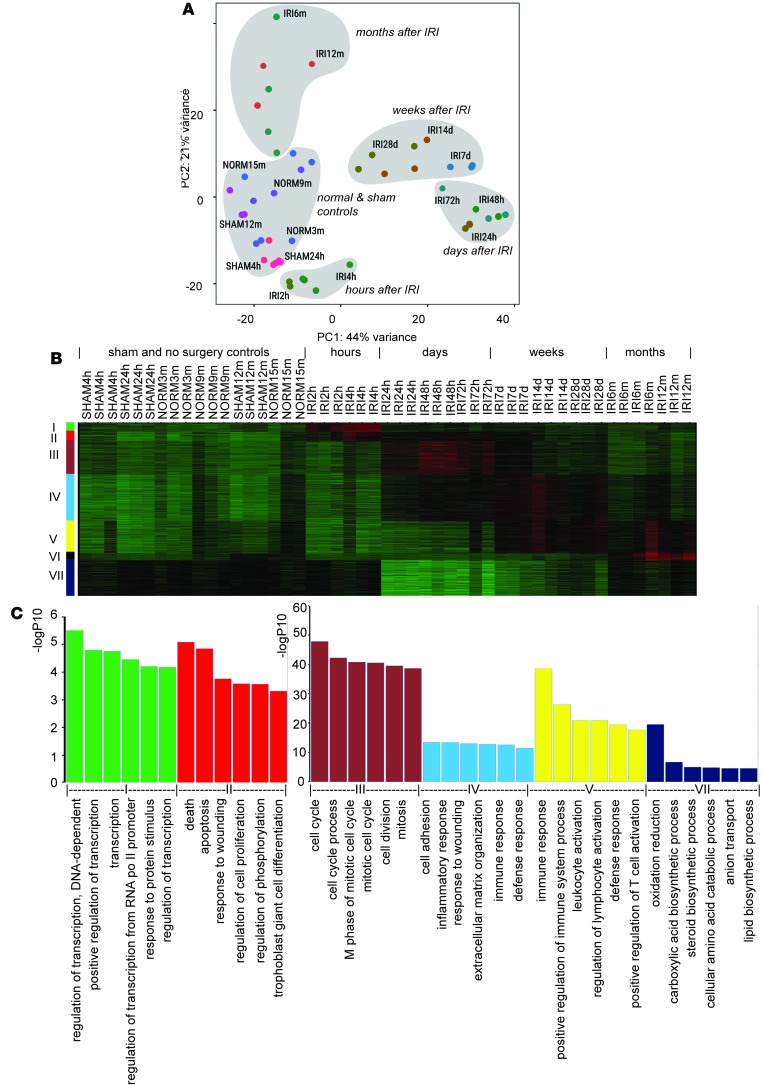
Temporal-specific gene changes after IRI through RNA-seq. (**A**) Sample clustering through principle component analysis (PCA) plot. (**B**) Heatmap of expression profiles of genes identified in modules with induction at hours (I), hours to weeks (II), days (III), days to weeks (IV), weeks to months (V), months (VI), and downregulation (VII). (**C**) Histogram of –log10 of *P* values of DAVID gene ontology for biological processes of module I~V and VII. SHAM4h, SHAM24h, and SHAM12m: 4 hours, 24 hours, and 12 months after sham surgery; NORM3m, NORM9m, and NORM15m: age-matched no surgery controls for 0, 6, and 12 months after IRI; IRI2h, IRI4h, IRI24h, IRI48h, IRI72h, IRI7d, IRI14d, IRI28d, IRI6m, and IRI12m: 2 hours, 4 hours, 1 day, 2 days, 3 days, 1 week, 2 weeks, 4 weeks, 6 months, and 12 months after IRI.

To dissect the kidney injury response at the molecular level and provide a comprehensive data resource for informing future injury studies, we performed whole-kidney total mRNA sequencing of 3 to 4 biological replicates at each time point (2 hours, 4 hours, 24 hours, 48 hours, 72 hours, 7 days, 14 days, 28 days, 6 months, and 12 months). Experimental injury samples were complemented by sham IRI surgery controls (4 hours, 24 hours, and 12 months) and age-matched nonsurgical controls (NORM3m, NORM6m, and NORM12m). Biological replicates clustered tightly together in sample clustering histogram, indicating a high degree of similarity in the temporal response to AKI in the injury model (Supplemental Figure 7A). Further, principle component analysis showed that the data clearly segregated into five distinct groupings (Figure 3A, denoted in gray). As expected, all sham and age-matched controls grouped together. In addition, distinct groupings were observed for responses after IRI in the hour, days, weeks, and months after IRI (Figure 3A).

To examine gene expression profiles in detail, we analyzed RNA-seq data at the gene level by quantifying fragments per kilobase of transcript per million mapped reads (FPKM) values from 43,309 genes. The full table containing FPKM values for all samples has been deposited in the NCBI Gene Expression Omnibus (GEO accession GSE98622). We identified 2,134 differentially expressed genes by comparing IRI and sham surgery samples in which the gene showed an FPKM value greater than 1 and a fold change greater than 4 for at least one of the time points with a FDR of less than 5%. Unsupervised hierarchical clustering of this gene set grouped differentially expressed genes into 7 modules of temporally modulated gene activity (Figure 3B and Supplemental Table 1).

To examine more directly the biological processes related to these genes, we performed DAVID gene ontology (GO) biological process enrichment analysis for each of the 7 gene sets. Redundant terms from enriched GO lists were removed by filtering out parent GO terms with 40% fewer genes than their child GO terms (43) (Supplemental Table 2). The top 6 terms for each module are shown in Figure 3C.

### Immediate early transcriptional responses after IRI are conserved between mouse and human.

The 101 genes in the first module (Figure 3B, green) were rapidly and transiently upregulated in the first hours after IRI. A comparison of the 101 genes in the early response module with our previous nephron-focused Six2-TRAP IRI_4h microarray samples identified that 53% of the genes were shared between both data sets. Given the nephron specificity of Six2-TRAP, much of the immediate early response localizes to nephrons.

The top three enriched GO terms in this module were exclusively related to transcription (Figure 3C). Indeed, over 20% of genes in this grouping encode transcriptional regulators, though transcription factors only comprise around 6% of genes in any tissue (44). Many of these transcription factors are known immediate early genes activated in response to a variety of external stimuli (45). The top overrepresented KEGG pathway was mitogen-activated protein kinase MAPK signaling pathway, and some of the enriched genes involved are as follows: AP1 family transcription factors (*Fos*, *Jun*, *Jund*); dual-specificity phosphatases (*Dusp1*, *Dusp2*, *Dusp5*, *Dusp8*, *Dusp14*); growth arrest and DNA damage-response genes (*Gadd45a*, *Gadd45b)*; ER stress–related gene *Ddit3* (also known as *Chop/Gadd153*); and apoptosis-related nuclear receptor *Nr4a1* (also known as *Nur77*).

**Figure 4:**
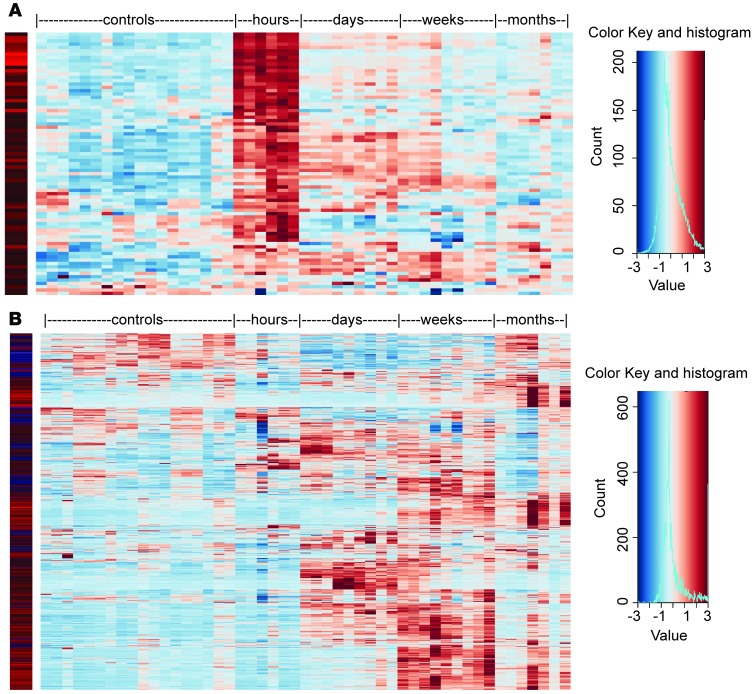
Overlap of mouse IRI with published human kidney transplant profiles. Heatmap of expression profiles in mouse IRI samples for differentially expressed genes identified in published human kidney transplant (**A**) 45–60 minutes (46) and (**B**) 1 year after reperfusion (74). Left columns indicate relative expression in human samples. Detailed sample information for each column is the same as that in Figure 3B.

To determine whether early mouse and human injury responses are similar, we compared the mouse data with published microarray expression profiles (GEO accession GSE43974) of human kidney transplant biopsies from deceased donors taken 1 hour after reperfusion (46) (Supplemental Table 3). The heatmap in Figure 4A shows the mouse IRI expression profiles of 84 upregulated genes identified from the human kidney transplant microarray study (46). Of these, 60.7% were strongly upregulated in the first few hours after IRI in the mouse model, indicating a strong conservation in the early injury response (hypergeometric test *P* < 0.01). Notably, 15 of the 21 transcription factors in the mouse early response module I (71.4%) were identified in human kidney graft biopsies: *JUN*, *FOS*, *BTG2*, *ZFP36*, *FOSB*, *EGR1*, *KLF4*, *KLF6*, *JUNB*, *CSRNP1*, *ATF3*, *JUND*, *NFIL3*, *DDIT3*, and *MAFF*. Other shared immediate early genes between mouse and human encode the growth factor, *HBEGF*; death-related genes, *BAG3*, *RHOB*, *NR4A2*, *PPP1R15A*, *GADD45B*; and heat shock protein family members *DNAJB1* and *DNAJB4*. Interestingly, a parallel comparison of published human liver transplants examining tissue samples collected 2–3 hours after reperfusion showed a significant overlap (hypergeometric test *P* < 0.01), with 13% of the 1,073 differentially expressed human liver genes upregulated in mouse kidneys 2–4 hours after IRI (47) (Supplemental Figure 7B and Supplemental Table 4).

Taken together, these data indicated that oxidative stress and hypoxia initiate a cascade of stress and immediate early transcriptional responses in the early phase of kidney injury that are largely conserved between mouse and humans and are commonly activated upon transplanting deceased donor kidneys. Further, some responding genes are more generally activated in other organ transplants.

### Persistent elevation of genes regulating cell death and proliferation from hours to weeks after IRI.

The 99 genes in module II (red, Figure 3A and Supplemental Table 1) showed rapid induction within hours, similar to genes in module I; however, enhanced expression was sustained for weeks after IRI. This group included the two injury markers discussed earlier: *Krt8*, *Krt20*, and *Sox9*, a transcriptional regulator recently linked to renal tubular repair programs (48, 49).

GO term enrichment analysis (Figure 3B) highlighted death and apoptosis-related terms and included genes encoding transcription regulators associated with cell death (*Gata6*, *Litaf*, and *Id1*) and cell proliferation (*Klf5*, *Fosl2*, *Zbtb16*, *Sox9*, *Myc*). A number of cytokine-encoding genes that showed extensive upregulation (*Lif*, *Cxcl1*, *Cxcl2*, *Il6*) and the receptor-encoding gene *Rtn4rl2* were enriched in Foxd1-TRAP analysis of interstitial cell types at 24 hours after IRI (28). *Rtn4rl2* (reticulon 4 receptor-like 2), also known as Nogo receptor homolog NgR2, is a member of Nogo66 receptor family (50). The Nogo signaling pathway has been reported to limit axon growth after injury (51), restrict synapse number in the developing hippocampus (52), and resolve inflammation in injured peripheral nerve (53). The very early onset and continuous upregulation of this group of genes is consistent with actions in the epithelial injury and repair process and with ensuing interstitial fibrosis.

### Genes related to cell cycle and wound repair are transiently upregulated days after IRI.

Module III (brown, Figure 3C and Supplemental Table 1) contained 371 genes with a transient high induction in the first 3 days, peaking around 2 days after IRI and returning to nearly basal levels by 1 week. This group included two ischemic/nephrotic AKI marker genes that dramatically increased at day 2: *Havcr1* and *Lcn2* (also known as *NGAL*) (54). Here, the top enriched GO terms were related to cell cycle, cell division, and mitosis (110 genes with *P* < 1E-40). Fifty genes are functionally annotated to M phase of the cell cycle, including *Ccnb1* (cyclin B1) and *Cdk1* (cyclin-dependent kinase 1). This finding is in line with the initiation of cell proliferation by proximal tubule cells surviving injury and expansion of interstitial fibroblasts in conjunction with renal fibrosis (16, 20).

Among the remaining 261 genes in module III, a second round of GO analysis revealed 15 genes related to wound repair (*Kng1*, *Arg1*, *Fgg*, *Dysf*, *Procr*, *Fga*, *Saa1*, *Fgb*, *Ccr1*, *F13a1*, *Saa3*, *Pf4*, *Entpd1*, *Orm2*, and *F2r)* and 15 genes related to cell adhesion (*Cldn7*, *Col13a1*, *Itga2*, *Itgb3*, *Emilin2*, *Col5a3*, *Cldn14*, *Nrcam*, *Lyve1*, *Itga5*, *Msln*, *Lamc2*, *Col8a1*, *Adam8*, and *Spp1*). Based on cell-specific TRAP microarray in AKI (28), we identified *Fga*, *Fgb*, and *Fgg* (fibrinogen α, β, and γ) production from nephrons; *Il11* (IL-11) production and elevated *Itga5* (integrin subunit α5) from interstitial cells; *Nrarp* (Notch-regulated ankyrin repeat protein) from endothelial cells; and *Retnlg* (resistin-like γ) from myeloid lineage cells. Taken together, the data suggest a complex interplay among multiple cell types for fibrosis, inflammation, and activated vasculature in the first few days after injury in addition to the marked reparative response within damaged nephron segments.

### Genes related to cell adhesion and inflammation were upregulated from days to weeks after IRI.

Module IV (turquoise; Figure 3C and Supplemental Table 1) is the largest with 516 genes that show a common temporal profile: upregulated from day 1 and elevated for weeks after IRI. This group included *Timp2* (tissue inhibitor of metalloproteinase 2), a cell cycle arrest biomarker for AKI (55, 56).

The top enriched GO terms in this group identified cell adhesion and inflammation as key cellular responses (141 genes with *P* < 1E-10). Among the well-documented fibrosis-related genes in this module are *Acta2*, *Col3a1*, *Timp1*, and *Pdgfrb*. Twenty-two genes are related to extracellular matrix organization: *Lgals3*, *Spock2*, *Mmp9*, *Olfml2b*, *Eln*, *Col3a1*, *Ccdc8080*, *Postn*, *Nid1*, *Serpinh1*, *Col5a1*, *Emilin1*, *Anxa2*, *Tgfb2*, *Smoc2*, *Fbln1*, *Crispld2*, *Tgfbi*, *Pdgfra*, *Bcl3*, *Lox*, and *Adamts2*. Eight genes are related to collagen-directed catabolic processes: *Prtn3*, *Mmp9*, *Mmp19*, *Mmp7*, *Mmp14*, *Mmp3*, *Adamts2*, and *Mmp2*. These data likely reflect active production and clearance of fibrosis-related extracellular products.

This phase correlates with the infiltration of inflammatory cells into renal tissue (see above). Consistent with this observation, module IV is enriched for genes associated with regulation of the complement system and Toll-like receptors (*Il18rap*, *Masp1*, *Il1rl1*, *C3*, *Il1rl2*, *Tlr13*, *Tlr2*, *Tlr7*, *Tgfb1*, *Tlr8*, *Cfp*, *Slc11a1*, *Tmem173*, *Cfh*, *Cfi*, and *Lbp*) and cytokines receptor signaling (*C3ar1*, *C5ar1*, *Ccl2*, *Cxcl5*, *Ccl9*, *Itgb2*, *Fpr2*, *Ccl7*, *Itgam*, *Fcgr3*, *Ccl6*, *Cxcl10*, *Tgfb2*, *Cxcl17*, *Ccl20*, *Robo1*, *Csf3R*, *Lbp*, and *Cmtm3*). Upregulation of *Nlrp3* indicates the potential for inflammasome activation and amplification of the inflammatory response (57), a deleterious outcome following renal ischemia (58). Interestingly, removing the 141 genes comprising the top (adhesion and inflammatory) GO terms and performing secondary GO analysis identified the Wnt receptor signaling pathway as the top GO term, including the following annotated genes: *Wnt2*, *Wnt10A*, *Dkk3*, *Sdc1*, *Wnt4*, *Nkd1*, *Nkd2*, *Ndp*, *Tgfb1l1*, *Axin2*, and *Cpz*. This finding is consistent with prominent linkage of Wnt signaling to renal fibrosis (59) and potentially to renal repair (60).

### Genes related to adaptive immune responses were identified starting from weeks to months after IRI.

Module V (yellow; Figure 3C and Supplemental Table 1) contained 352 genes that were upregulated starting from week 1 to months after IRI. The top enriched GO terms identified 101 genes that were exclusively related to immune responses, including leukocyte activation and regulation of lymphocyte activation, including *Ptprc*, *Itgal*, *Ikzf1*, *Cd3d*, *Cd3e*, *Il7r*, *Was*, *Vav1*, *Fkbp1b*, *Cd74*, *Cd48*, *Card11*, *Dock2*, *Bcl2a1d*, *P2rx7*, *Cd86*, *Itgax*, *Lck*, *Zap70*, *Cd4*, *H2-dma*, and *Spn*. Of the 19 transcription factors enriched in this group (*Akna*, *Ankrd6*, *Batf*, *Ciita*, *Foxj1*, *Ifi16*, *Ikzf1*, *Irf4*, *Lpxn*, *Lyl1*, *Meis3*, *Nfkbie*, *Nlrc5*, *Pou2f2*, *Sox8*, *Spi1*, *Spib*, *Tcf7*, and *Vav1*), several encode lymphocyte lineage-related transcription factors: *Foxj1* suppresses spontaneous T cell activation and autoimmunity (61); *Spi1* (also known as Pu.1) and *Spib* regulate B cell differentiation (62); *Akna* regulates CD40 and CD40 ligand, costimulatory molecules for B and T cells (63); and *Ciita*, which encodes a master transcription factor for major histocompatibility complex II expression on antigen-presenting cells, including dendritic cells, B cells, and macrophages (64).

Among the enriched genes encoding cytokines and growth factors are *Aif1* (allograft inflammatory factor), originally identified in chronic rejection of rat cardiac allografts (65), an important player in autoimmunity (66); *Ccl22*, which is reported to prevent rejection of mouse islet allografts by recruiting Tregs (67); *Scube1*, encoding a secreted regulator of HH signaling that has been linked to renal IRI (68); and *Igfbp2*, a modulator of IGF and integrin signaling that has been reported as a potential biomarker for renal function and renal pathology in type II diabetes (69) and lupus nephritis (70). A second round of GO analysis on the remaining 251 genes uncovered 23 genes related to cell adhesion in the most enriched GO term: *Parvg*, *Siglece*, *Cadm3*, *Plek*, *Fermt3*, *Cpxm2*, *Ly9*, *Itga4*, *Ajap1*, *Vcam1*, *Podxl2*, *Itgb7*, *Cpxm1*, *Pstpip1*, *Mfap4*, *Gpnmb*, *Cd6*, *Negr1*, *Selplg*, *Col8a2*, *Klra2*, *Thbs4*, and *Dpt*.

### B cell–related genes were identified months after IRI.

Module VI (black; Figure 3C and Supplemental Table 1) comprised 82 genes upregulated at 6 and 12 months after IRI. These displayed an overwhelming signature with 83% (69 genes) of immunoglobin LV and V genes in module VI, consistent with our observations of CD45R^+^ cells in regions with severe interstitial nephritis (Figure 1, L–O).

### Genes related to tubular function were downregulated after IRI.

Module VII (blue; Figure 3A and Supplemental Figure 1) contained 405 genes markedly downregulated on IRI with a strong linkage to genes associated with normal metabolic processes in the kidney. A comparison with a published RNA-seq data set of the segmental profile of the adult rat nephron (71) identified 34 genes with endogenous expression in the proximal tubules (Table 1 and Supplemental Table 6), including *Kap* (kidney androgen-regulated protein) and *Lrp2* (Megalin, low density lipoprotein receptor–related protein 2). Most of these genes are highly expressed in the proximal tubule of the nephron and are functionally associated with tubular biosynthetic and metabolic processes. Module VII genes also contained genes with high basal expression in the collecting duct and loop of Henle, including *Slc4a1,* a Cl^–^/HCO_3_^–^ transporter localized to the cortical and outer medullary collecting duct (72); *Insrr*, an acid/alkali-sensing receptor (73) in cortical collecting ducts; and *Slc12a1* and *Ppp1r1a*, which are present in the thick ascending limb.

### Shared immune signaling pathways between mouse IRI and human 1-year kidney transplants.

To determine how well the long-term mouse IRI model mirrored human clinical cases, we examined the mouse ortholog expression profiles for differentially expressed human genes identified from published microarray (GEO accession GSE22459) on biopsies from 1-year kidney grafts with inflammation and fibrosis (74). The heatmap in Figure 4B showed distinct clusters of gene sets, with specific temporal induction reflecting common molecular changes during the progression of inflammation with fibrosis between mouse IRI and human kidney transplants. Of the 1,006 genes identified as differentiated in human data set (Supplemental Table 5), 575 (57.2%) of their mouse orthologs had fold change >1.5, FPKM >1, and FDR <5% in mouse IRI. The top overrepresented DAVID KEGG signaling pathways for the 575 genes were as follows: chemokine; cytokine-cytokine receptor interaction; natural killer cell–mediated cytotoxicity; Toll-like receptor; T cell receptor; NOD-like receptor; and B cell receptor signaling pathways. Of the 352 mouse genes uncovered in module V (yellow), most were genes associated with adaptive immune response and were upregulated in the weeks and months after IRI. However, 141 genes (40.5%) from this module were differentially expressed in 1-year human kidney transplants that displayed inflammation and fibrosis. In consideration of the multifactorial events that can influence renal function after kidney transplantation (including drug toxicity, infections, and subsequent AKIs), we had expected that the effect of the initial I/R injury was not detectable in the long term. However, this partial overlap between the transcriptional profile in our mouse model and that in kidney transplant biopsies suggests that the initial injury might be more relevant than expected even in the long term or that following episodes of renal injury in transplant recipients might trigger similar transcriptional responses. These observations are in line with recent studies suggesting a critical role of genes involved in kidney repair for the long-term outcome after kidney transplantation (75).

### Temporal and spatial renal tubular and inflammatory responses visualized by RNA section in situ hybridization.

**Figure 5:**
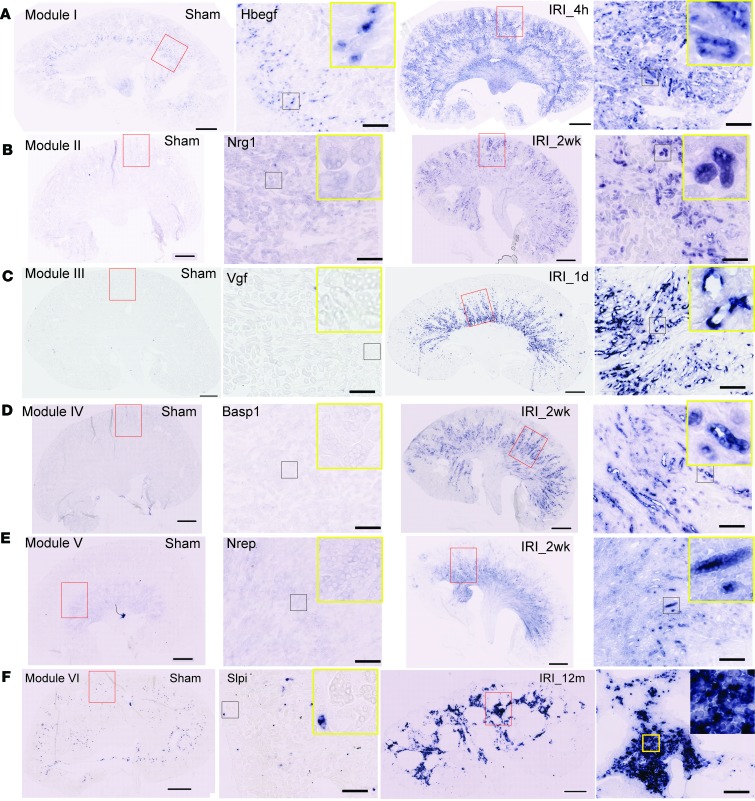
Expression patterns of representative genes from module I–VI through RNA section in situ hybridization. Expression patterns of representative genes from module I (**A**), module II (**B**), module III (**C**), module IV (**D**), module V (**E**), and module VI (**F**). Images in the second and fourth columns are high-magnification views of regions in red rectangles. Images in insets are high-magnification views of regions in black squares (the second and fourth columns in **A**–**E** and second column in **F**) and the region in the yellow square (the fourth column in **F**). Scale bar: 1 mm (whole-kidney view); 200 μm (zoomed view for red rectangular regions); 120 μm (insets).

To corroborate the quantitative results with RNA-seq, we selected one gene from each module for a detailed analysis of its temporal and spatial expression profile by in situ hybridization (Figure 5). *Hbegf* (heparin-binding EGF-like growth factor), a growth factor encoding gene and representative module I gene showed endogenous expression in medullary collecting ducts, while a robust increase was seen in cortical proximal tubules 4 hours after IRI. This is consistent with the protein upregulation in a previous published rat IRI model (76). In addition, increase of *Hbegf* mRNA was also seen in the loop of Henle adjacent to vasa recta in the medulla and a subset of papillary cells (Figure 5A). *Nrg1* (neuregulin 1), a glycoprotein-encoding gene regulating cell signaling in development and adult brain (77), within module II was localized in cortical proximal tubules 2 weeks after IRI (Figure 5B). *Vgf* (VGF nerve growth factor inducible), a gene in module III, encodes a neuronal and endocrine peptide precursor that is normally expressed in nerve system, adenohypophysis, adrenal medulla, gastrointestinal, tract, and pancreas (78). The mRNA of *Vgf* was not detected in normal kidney but displayed strong, transient expression in the medullary thick ascending limb at day 1 after IRI (Figure 5C). *Basp1* (brain abundant, membrane attached signal protein 1), encoding a membrane attached signaling protein, within module IV was originally identified as a transcriptional corepressor of Wt1 (Wilms’ tumor) (79) that promoted apoptosis in diabetic nephropathy (80). RNA in situ exhibited its expression in the atrophied tubules in the outer medulla 2 weeks after IRI (Figure 5D). *Nrep* (neuronal regeneration-related protein), encoding an intracellular protein linked to the augmentation of TGF-β–induced renal fibrosis (81), within module V was active at day 14 after IRI in inner medullary tubules (Figure 5E). *Slpi* (secretory leukocyte peptidase inhibitor), which encodes a peptidase inhibitor regulating immunity and tissue regeneration (82) and module VI representative, was associated with the immune cell infiltrate in kidneys 1 year after IRI (Figure 5F). *Mfsd2a* (major facilitator superfamily domain containing 2A), a newly identified transporter for fatty acids specifically in brain-blood barrier (83, 84) and module VII representative gene, was markedly downregulated within its normal site of expression in the S3 segment of LTL^+^ proximal tubule (Supplemental Figure 6).

## Discussion

This study provides a long-term comprehensive histological, cellular, and molecular characterization of temporal-specific biological events following a single ischemic AKI in the adult mouse. Following a sublethal IRI, renal function was restored in 2 weeks, but molecular and cellular characterization reveal nephron loss and disrupted kidney programs that result in a severely compromised kidney over the course of 1 year. This study highlights progression from a single episode of ischemic AKI to acute renal dysfunction with histological features of end-stage renal disease by 1 year, suggesting that with a severe initial hit, multiple episodes of AKI are not essential for the progression of AKI to end-stage renal disease (85). These results provide a strong foundation for future studies on maladaptive repair mechanisms and key regulators for AKI-to-CKD transition.

Our cellular characterization of proximal tubule injury extends recent observations on the de novo activation of a number of keratin genes normally expressed within other nephron segments into injured proximal tubule cells. In particular, we identify *Krt20* as a marker of the injury response that is closely associated with well-characterized injury marker Havcr1. Krt20 and Krt8/18 are rapidly activated on injury in epithelial cells in the proximal tubule, and post-injury repair (7 days after IRI) remains in nonepithelial cells in degenerate proximal tubules.

Of particular note, the injury, repair and long-term maladaptive responses are underpinned by coordinated regulation of distinct gene sets. The immediate response to injury is dramatic upregulation of an array of transcriptional regulatory factors. Although much of the initial transcriptional response is transient, there is a subset of early activated genes that maintain expression from hours to weeks following IRI, presumably directed by persistent transcriptional control. Among these is Sox9, a transcription factor linked to proximal tubule repair processes and to unresolved injury that remains in damaged proximal tubule segments (48, 49). In a urothelial injury model, Sox9 induction resulted from activation of the epidermal growth factor receptor (EGFR) signaling cascade (86). Activation of the EGFR pathway has been implicated in CKD (87) as well as in diabetic kidneys (88). Notably, we identified 3 genes encoding EGFR ligands markedly upregulated in our AKI-to-CKD transition model: immediate early transient expression of the heparin-binding epidermal growth factor, *Hbegf*, and more persistent elevation of *Areg* (amphiregulin) and *Nrg1* (neuregulin 1). In particular, AREG has been reported recently to be upregulated in the urine of AKI and CKD patients and correlated with fibrosis (89). Unraveling the molecular basis of the network of EGF family ligands may provide insight into the initiation and progression of proximal tubular cell repair.

Besides the genes encoding well-characterized biomarkers, such as *Havcr1*, *Lcn2* and *Timp2,* RNA-seq identified a series of genes encoding neural signaling molecules upregulated after IRI. While some of these were transiently induced within days, such as the neuropeptide-encoding genes *Grp* (gastrin-releasing peptide) and *Vgf* (VGF nerve growth factor inducible), others were elevated for weeks, such as the growth factor–encoding gene *Ngf* (nerve growth factor) and extracellular matrix molecule encoding gene *Agrn* (agrin). Ngf has been reported in the serum of kidney transplant recipients at 6 months (90), while AGRIN, a major heparan sulfate proteoglycan product of motor neurons, is normally synthesized within the glomerulus and incorporated into the glomerular basement membrane (91). The c-terminal fragment of AGRIN has been proposed as an indicator for renal function in sepsis (92), a biomarker for kidney transplants (93), and an indicator of progression of kidney disease in type 2 diabetics (94).

Kidney transplantation begins with IRI, but there are obvious relevant differences between our mouse model and the clinical setting of organ donation. Despite considerable effort profiling human kidney transplant biopsies (46, 74, 75, 95, 96), the chronic effects caused by ischemia during kidney retrieval, transport, and implantation (97–100) on the long-term survival of renal allograft are unclear (101, 102). Though there is a paucity of comparable human RNA-seq data, we found a substantial overlap of gene sets indicative of immediate early responses after reperfusion comparing our mouse IRI model with published microarray data from deceased donor transplants of human kidneys (46) and of later fibrosis-associated responses documented in biopsies of human kidney transplants with immune associated fibrosis 1 year after transplant (74). The overlapping gene sets in mouse-human comparisons provide a focus for targeted follow-up of these cohorts. Further, the overlap suggests additional cross species relevance where human clinical data is not available for comparable analysis. Together, the well-validated databank will be a rich source of information for exploring kidney injury and its imperfect resolution.

## Methods

### IRI.

Warm renal ischemia-reperfusion injury was performed on 10- to 12-week-old (25–28 g) C57BL/6CN male mice from Charles River. All the mice received an intraperitoneal injection of a ketamine/xylazine mix (105 mg ketamine/kg; 10 mg xylazine/kg), and body temperature was maintained at 36°C throughout the procedure. Midline abdominal incision was performed for the exposure of both renal pedicles, which were clamped for 21 minutes using nontraumatic microaneurysm clips (Roboz Surgical Instrument Co.). Occlusion of blood flow was monitored by color change from normal to dark purple immediately after the clamping; blood returned to its normal original color after removal of the clamps. Mice underwent the same procedure; mice with clamping of the pedicles were used as sham surgery controls.

### RNA-seq.

RNA samples were provided to the University of Southern California Epigenome Center Data Production Core Facility for library construction and sequencing. RNA integrity was verified by Bio-Rad Experion analysis. Library construction was carried out using the Illumina TruSeq RNA Sample Prep kit v2 through polyA selection. The manufacturer’s protocol was followed with the exception that the final PCR amplification was performed for 12 and not 15 cycles. Libraries were visualized on the Agilent Bioanalzyer and quantified using the Kapa Biosystems Library Quantification Kit according to manufacturer’s instructions. Libraries were applied to an Illumina flow cell at a concentration of 16 pM on a version 3 flow cell and run on the Illumina HiSeq 2000 as a paired end read for 100 cycles each side. On average, 3 samples were loaded to each lane of a flow cell. Image analysis and base calling was carried out using RTA 1.13.48.0. Final file formatting, demultiplexing, and fastq generation were carried out using CASAVA v 1.8.2.

### Bioinformatics analysis.

RNA-seq samples were aligned to mm10 genome assembly with STAR aligner (version 2.5.0b) (103). The mapping index was generated using GENCODE release M4 (GRCm38.p3) gene annotations (104). In addition to read mapping, STAR was also used to remove duplicates, generate read-count tables, and wiggle files. Differentially expressed genes were called for each time point with DESeq2 (105). For all the analyses, we only kept genes with (a) FDR-transformed *P* values below 0.05, (b) log fold change of at least 2, and (c) FPKM above 1 in IRI and/or control samples. These values of log fold change and FPKM thresholds were chosen to enable experimental validation of our differential-expression calls. Differentially expressed genes were separated into groups according to their expression profile similarity as follows. We first transformed the counts using variance-stabilized transformation in DESeq2 and then applied the WGCNA R package (106). WGCNA analysis was performed using the blockwise modules function and signed correlation networks selected. Power 30 was chosen for soft thresholding according to scale-free topology criterion. The minimum coexpression module size was set to 10, and merge cut parameter was set to 0.25.

### Serum and urine analysis.

Serum creatinine levels were measured at the UT Southwestern Medical Center O’Brien Center for Kidney Disease Research by capillary electrophoresis (PA800 Plus Pharmaceutical Analysis System, Beckman Coulter). BUN was assayed on the Stanbio Excel analyzer using BUN Procedure 0580 for the quantitative colorimetric determination of urea nitrogen in serum. Urine albumin was assayed using the Mouse Albumin ELISA Quantitation Set (catalog E90-134, Bethyl Laboratories) on the Bio-Rad microplate reader. Urine creatinine was assayed using the QuantiChrom Creatinine Assay Kit (BioAssay Systems). BUN and urine assays were performed at George M. O’Brien Kidney Center at Yale University.

### Histology.

Kidneys were perfused with ice-cold PBS and embedded in paraffin after overnight fixation in 4% paraformaldehyde at 4°C. Sections were cut at 5 μm and stained with H&E. Tubular injury was scored with H&E-stained sagittal sections of whole kidney semiquantitatively with the following criteria: 0, no tubular injury; 1, <10% tubules injured; 2, 10%~25% tubules injured; 3, 26%~50% tubules injured; 4, 51%~75% tubules injured; 5, >75% tubules injured (107). 20 fields (10 in cortex and 10 in medulla) at ×20 magnification were examined for tubular damage, defined as necrotic, dilated, atrophied tubules and tubular casts. Tubular cystic formation was quantified by the percentage of cystic epithelial area in scans of the kidney using ImageJ (NIH). Glomerular sclerosis was assessed by the percentage of the number of glomeruli with mesangial hypercellularity feature in the H&E-stained kidney sagittal sections. Trichrome staining was performed at the University of Southern California Norris Cancer Center Translational Pathology Core Facility. Fibrosis was assessed semiquantitatively by the ratio of collagen deposition area in trichrome-stained sagittal sections (blue) over the whole field in multiple cortical and medullary fields through ImageJ.

### Immunofluorescence.

Paraformaldehyde-fixed tissues were equilibrated in 30% sucrose/PBS overnight and then embedded in OCT within a dry ice ethanol bath. Eight- to ten-μm frozen sections were washed in PBT (PBS + 2.5% Triton-X), blocked in 5% normal donkey serum in PBT, and incubated overnight at 4°C with primary antibodies before incubating with species-specific secondary antibodies coupled to Alexa Fluor dyes (488, 555, 594, and 647; Life Technologies) for 1 hour at room temperature. The following antibodies were employed in this study recognizing Havcr1 (kidney injury molecule-1, goat, 1:1,000, R&D Systems, AF1817), α-SMA (Cy3, mouse monoclonal, Sigma-Aldrich, C6198), CD3 (rabbit, Abcam, ab16669); and CD4 (rat, BD Pharmingen, 550280). CD45 (goat, R&D Systems, AF114). CD19 (rat, Thermo Fisher Scientific, 13-0194-82), F4/80 (rat, eBiosciences, 14-4801), CD45R (rat, BD Pharmingen, 557390), cytokeratin 20 (rabbit, Abcam, ab118574), cytokeratin 8/18 (rabbit monoclonal, Abcam, ab53280; rat supernatant, DSHB, troma-1), PECAM1 (rat, 550274, BD Pharmingen), LTL lectin-FITC conjugate (Vector Laboratories, FL-1321), biotinylated LTL (Vector Laboratories, B-1325), and desmin (mouse, DAKO, M0760). All sections were stained with Hoechst 33342 (Life Technologies) prior to mounting with Immu-Mount (Fisher). Fluorescent images were acquired on a Zeiss Axio Scan Z1 slide scanner and Zeiss LSM780 and Leica SP8 confocal microscopes. Vessel density was quantified by the number of PECAM1^+^ vessels that intersected with an overlaid 12 × 12 gridlines (108). 20 fields for each section were randomly selected (10 in cortex with glomerulus excluded and 10 in outer medulla) in the sagittal sections of whole kidneys.

### RNA section in situ hybridization.

Eight- to ten-μm frozen sections were incubated overnight at 4°C in 4% paraformaldehyde, and in situ hybridization was performed using digoxigenin-labeled (DIG-labeled) antisense riboprobes according to a previously published protocol (28). For riboprobes of *Vgf* and *Slpi*, synthetic cDNA fragments with T7 promoter at 3′ end of the reverse strand were used for DNA template. RT-PCR products with primer pairs for *Hbegf* (forward: acttggaagggacagatctgaac; reverse: taatacgactcactataggggctctcttccagtccataaacca); *Nrg1* (forward: gttatttctctggatccaacggc; reverse: taatacgactcactatagggtgtagtgactggtggaaaaggag); *Basp1* (forward: ggcttcagactctaaacctagca; reverse: taatacgactcactatagggggttggcattgagatacatgtgg); *Nrep* (forward: ggaagggagaatagaagaagccc; reverse: taatacgactcactatagggccgacaccttcttagaacatgga), *Mfsd2a* (forward: ccacttcctgcttccattatcct; reverse: taatacgactcactataggggtccaaggtataggtgcagaaca) were used for DNA templates for riboprobes. Fixed sections went through permeabilization (Proteinase K, 10 μg/ml, 20 minutes), acetylation (0.375% acetic anhydride), and hybridization (overnight, 68°C) with 0.5 μg/ml riboprobe in hybridization buffer (50% formaldehyde, 5× SSC, 1% SDS, 50 μg/ml yeast tRNA, 50 μg/ml heparin). After RNase treatment (2 μg/ml, 15 minutes, 37°C), sections were incubated with anti-DIG-AP antibody (1:4,000, Roche, 11093274910, 4°C, overnight) and developed with chromogenic substrate (BM Purple, Roche) for 4 hours to 7 days until a sufficient staining intensity was reached. Sections were fixed in 4% paraformaldehyde again and then either directly mounted with Glycergel (Dako) or stained with FITC-LTL before mounting. Sections were imaged on a Zeiss Axio Scan Z1 slide scanner.

### Statistics.

Values for serum creatinine, BUN, percentage of urine albumin/creatinine, percentage of cyst area, and percentage of number of sclerotic glomeruli were represented as dot plots and mean ± SEM. The significance of difference among groups was examined using unpaired 2-tailed *t* test. A *P* value of less than 0.05 was considered significant. Tubular injury score, percentage of trichrome blue area, and percentage of vessel density were presented as mean ± SEM. All analyses were based on 3 independent repeats of experiments with 3–17 biological replicates.

### Study approval.

All surgical procedures, mouse handling, and mouse husbandry were performed according to guidelines issued by the Institutional Animal Care and Use Committees (IACUCs) at the University of Southern California (protocol 11911) and were performed after obtaining approval from the University of Southern California’s IACUC.

## Author contributions

JL, SK, and APM contributed to conception and experimental design; SK, JL, MCD, and AMK performed IRI surgeries; JL, SK, ED, MCD, YC, ML, ADS, GFA, GS, PEC, RKP, JG, and CL performed data acquisition or data analysis; and JL and APM prepared the manuscript, incorporating comments from other authors.

## Supplementary Material

Supplemental data

Supplemental figures

## Figures and Tables

**Table 1:**
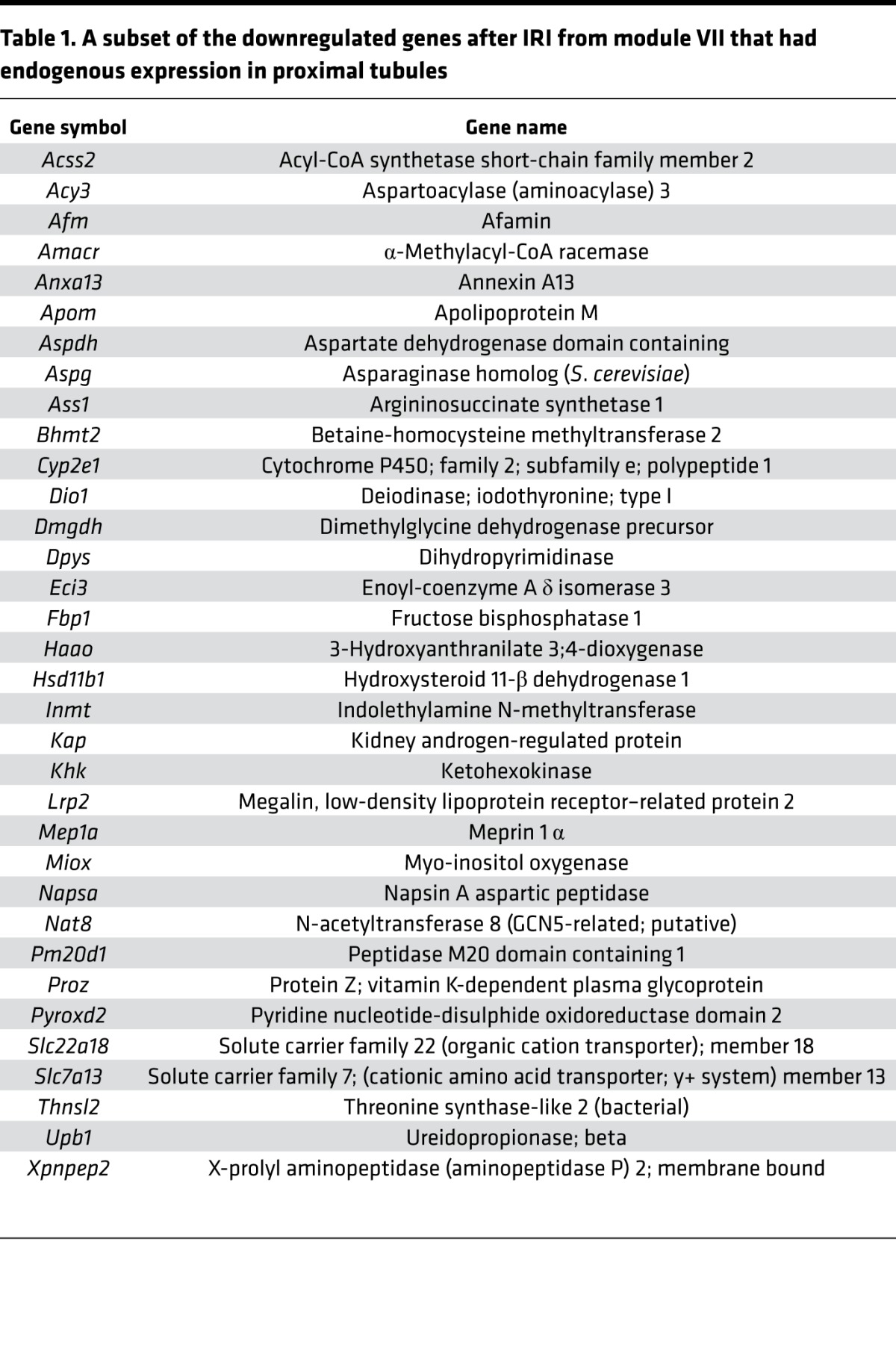
A subset of the downregulated genes after IRI from module VII that had endogenous expression in proximal tubules

## Version 1 09/14/2017

Electronic publication

## Version 2 09/25/2017

Updated supplemental data files.
