# Supplementary material for: Molecular characterization of the transition from acute to chronic kidney injury following ischemia/reperfusion
Source: JCI Insight. 2017 Sep 21;2(18):e94716. doi: 10.1172/jci.insight.94716 (PMC5612583; doi:10.1172/jci.insight.94716)

Supplemental Figure 1: Incomplete recovery following bilateral renal IRI. Dot plots for (A) Serum Creatinine (mg/dl), (B) BUN (mg/dl), (C) Percentage of urine albumin(mg/dl) vs urine creatinine (mg/dl), (D) Percentage of the area of cysts per whole kidney middle sagittal section, (E) Percentage of the number of sclerotic glomeruli in sham 6 months, IRI 6 months and IRI 12 months kidneys. Mean was presented. * Difference is significant between groups with P value <0.05. (F) Histogram of tubular injury score. Data was presented as mean ± SEM. (G) - (L) Zoomed in view of the rectangle regions in the cortex and outer stripe of outer medulla of HE stained kidneys in Figure 1(D-I). Scale bar 50µm.

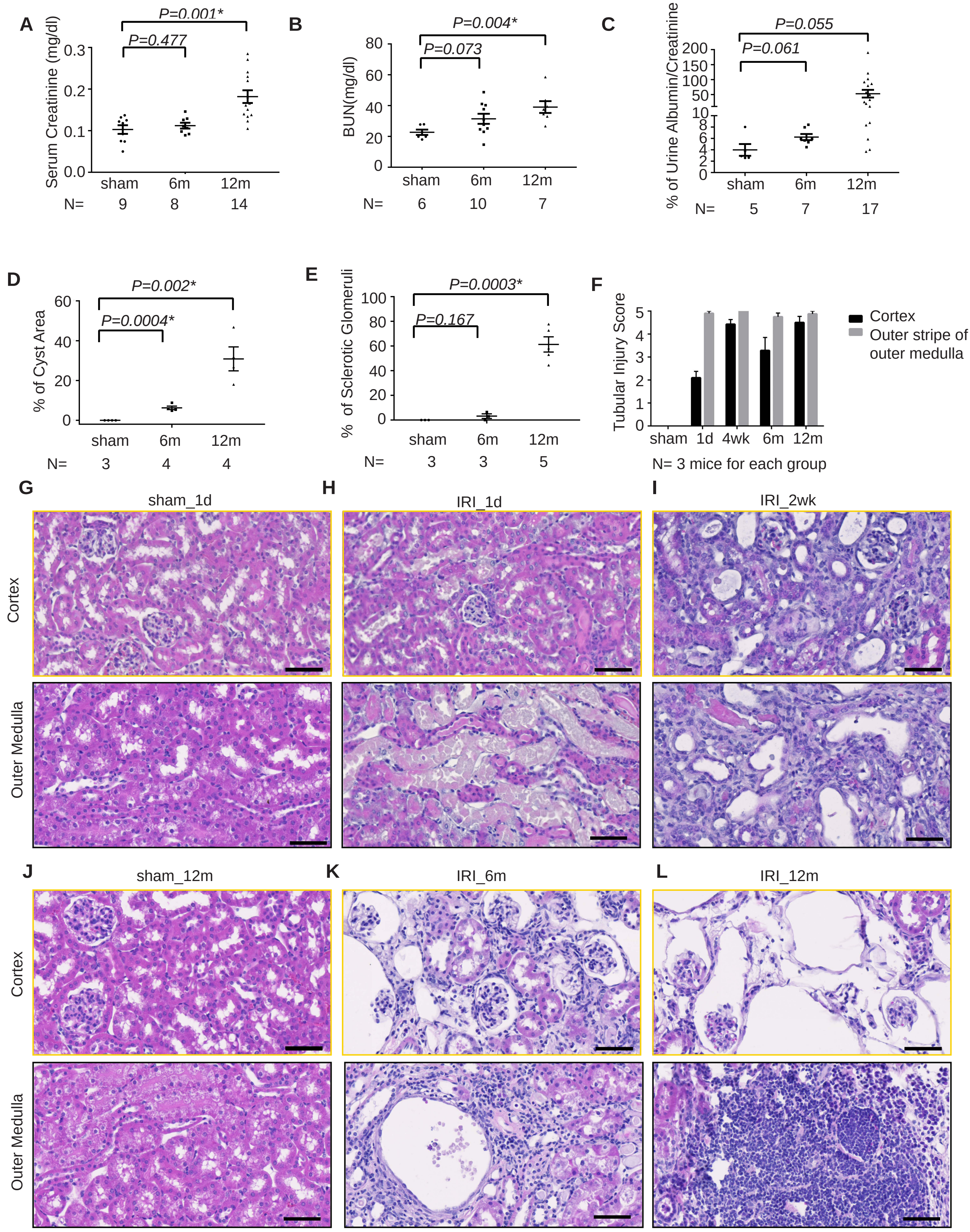

Supplemental Figure 2: Interstitial fibrosis in mouse bilateral renal IRI model. Trichrome staining on kidneys sham 12 months (A, F); IRI 1 day (B, G); IRI 2 weeks (C, H); IRI 6 months (D, I) and IRI 12 months (E, J). Whole kidney view (A-E) and zoomed in view of the squares in cortical (yellow), outer (blue) and inner (black) medullary regions (F-J). Scale bar 1mm (A-E), 50µm (F-J). (K) Histogram of the percentage of the blue color area on the trichrome stained kidneys quantified by ImageJ. Data was presented as mean ± SEM.

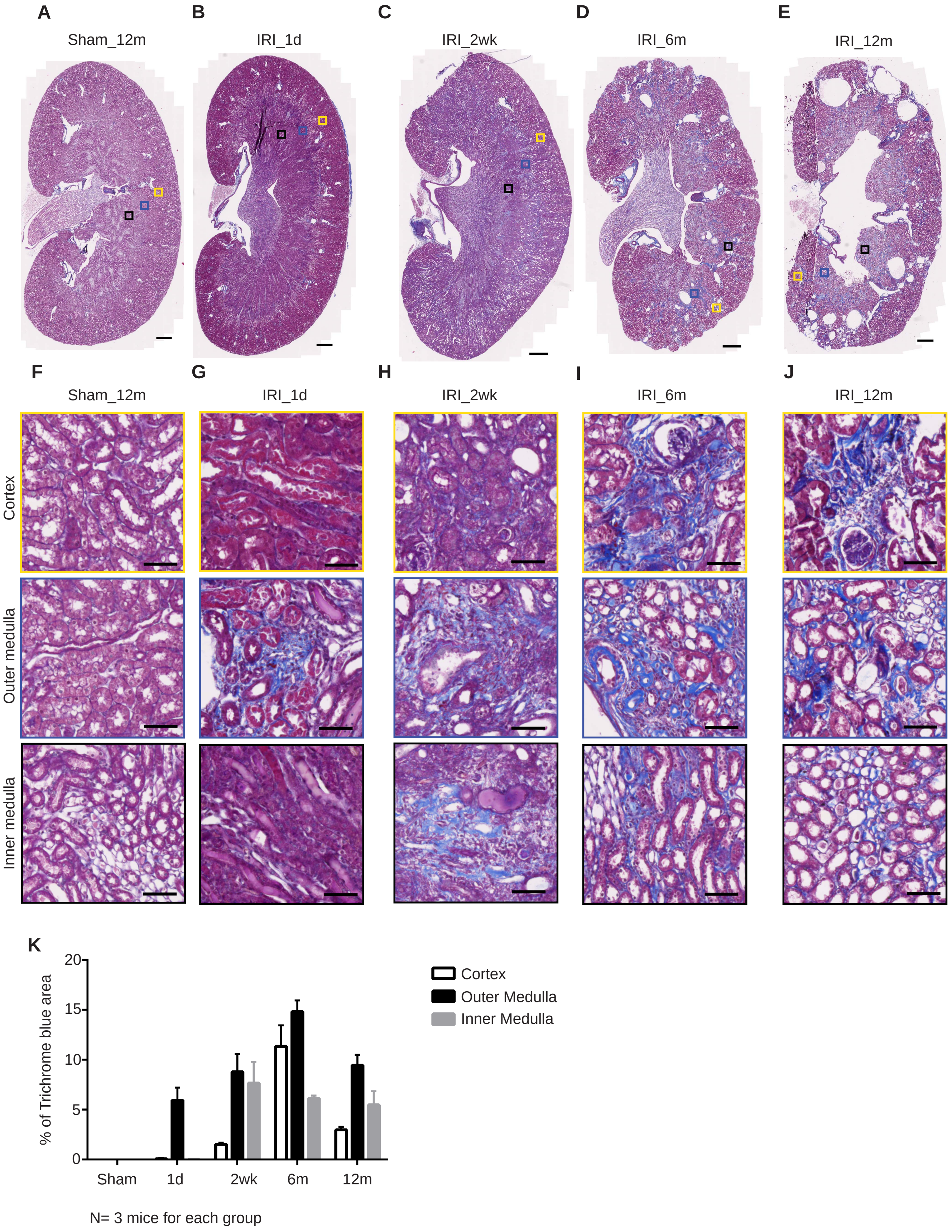

Supplemental Figure 3: Cytokeratin expression in injured kidneys. (A) Histogram of six2-TRAP microarray fold changes (FC) IRI 4 hours and IRI 24 hours vs Sham 24 hours for cytokeratin with FC>2, FDR<0.05. Data was presented as mean ± SEM. Whole kidney (B, C) and zoomed in view of the yellow rectangle regions (D, E) on sagittal sections of kidneys 4 weeks post sham (B, D) and IRI (C, E) with immunostaining of Krt20(red) and α-SMA(cyan). Confocal immunofluorescence of Krt20(red), Havcr1(Kim1) (green), LTL(cyan) in the cortex on sagittal sections of kidneys 3 days (F) and 4 weeks (L) post sham; 3 days (G), 1 week (H) and 4 weeks (M) post IRI. Merged images (F, G, H, L, M) and split channels (I, J, K, N, O). Scale bar 1mm (B,C), 50µm (D-O).

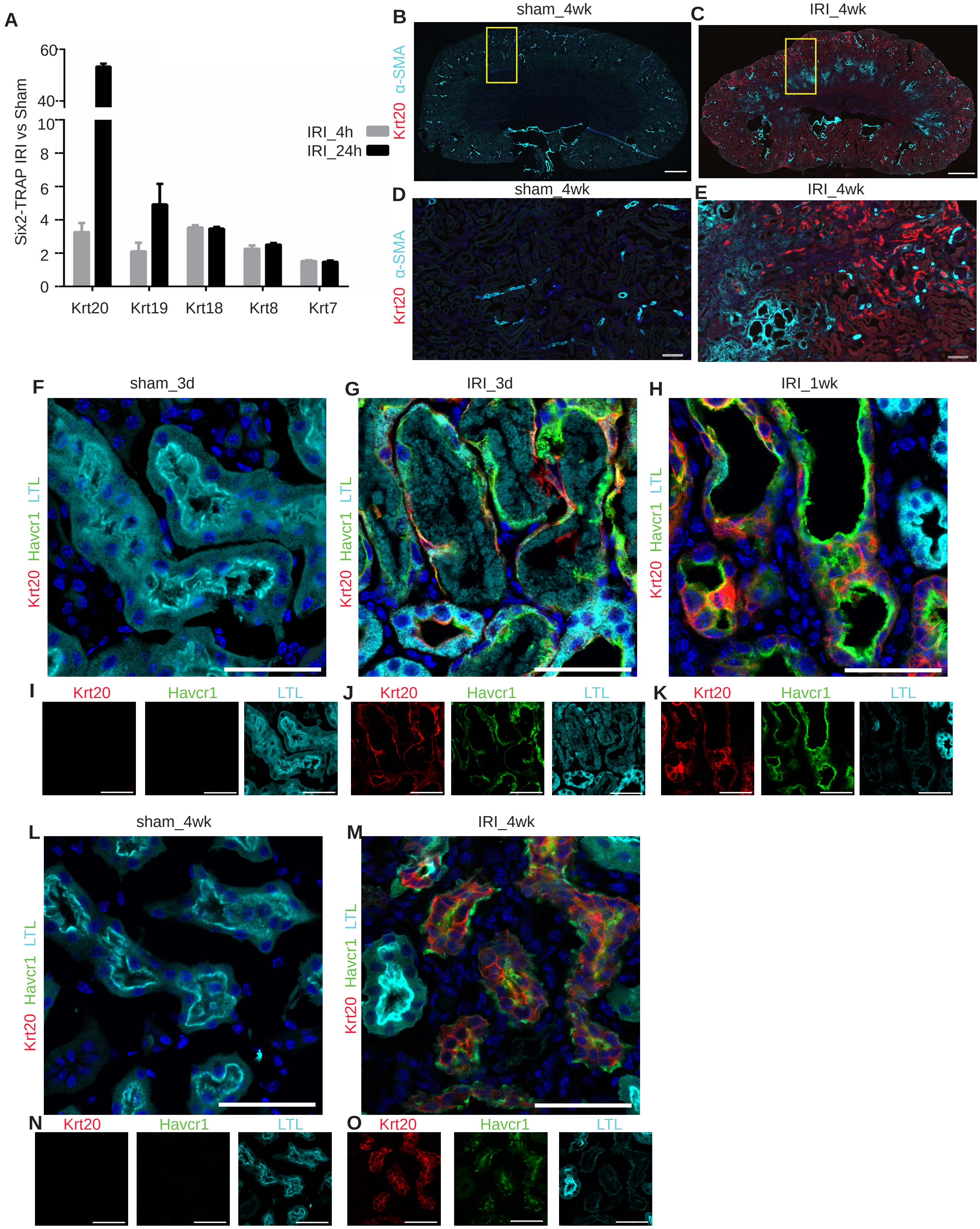

Supplemental Figure 4: Capillary loss in IRI kidneys. Whole kidney view of sagittal sections of kidneys(A-C) and zoomed in view of confocal immunofluorescence 3 days post sham(A, D), 3 days post IRI (B, E) and 2 weeks post IRI (C, F) with immunostaining of Havcr1(Kim1)(cyan), PECAM1(CD31)(red), LTL(green). Scale bar 1mm (A-C), 50µm (D-F). (G) Histogram of the percentage of vessel density in IRI kidneys vs Sham. Data was presented as mean ± SEM.

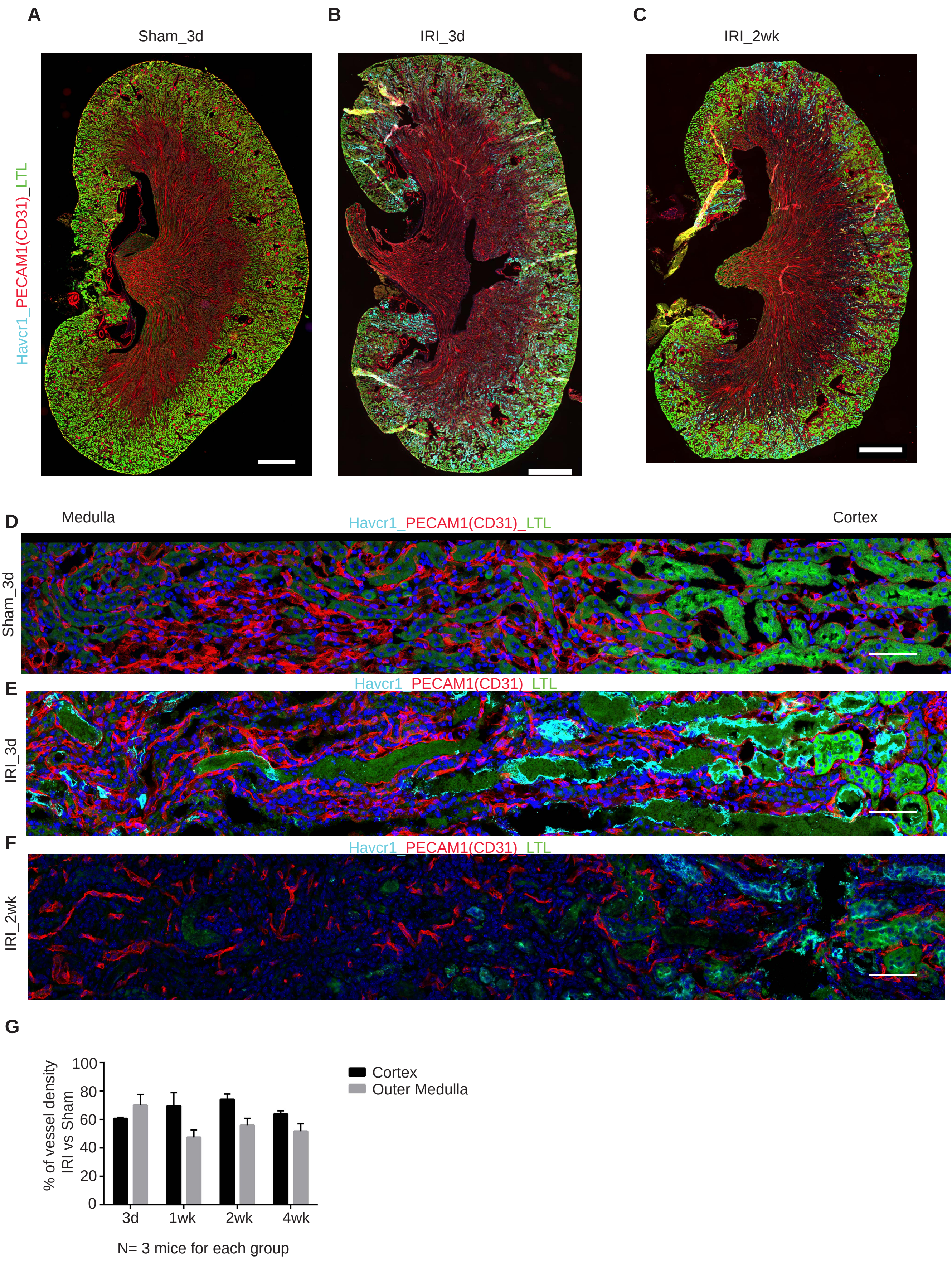

Supplemental Figure 5: Immune cell infiltration in IRI kidneys. Whole kidney view of sagittal sections of kidneys 2 weeks post sham(A) and IRI (B) with immunostaining of CD4(green), α-SMA(red) and Desmin (green). Zoomed in view of confocal immunofluorescence for F4/80 (yellow), Krt20(magenta) and LTL (green) from yellow rectangle region (C, sham) and (D, IRI). Zoomed in view of confocal immunofluorescence for CD4(green), α-SMA(red) and Desmin (green) from magenta rectangle region (E, sham) and (F, IRI). Scale bar 1mm (A-B), 50µm (C-F). (G) Histogram of the percentage of CD45+ cells among all kidney cells on sagittal sections of kidneys fro sham, 3d, 1 week, 2 weeks, 4 weeks 12 months post IRI. Subsets of immune cells were quantified by the percentages CD45 positive cells that were F4/80 and CD45 double positive(yellow); CD3, CD4 and CD45 triple positive(blue); CD45R and CD45 double positive (red) and CD45 single positive cells (purple). Data was presented as mean.

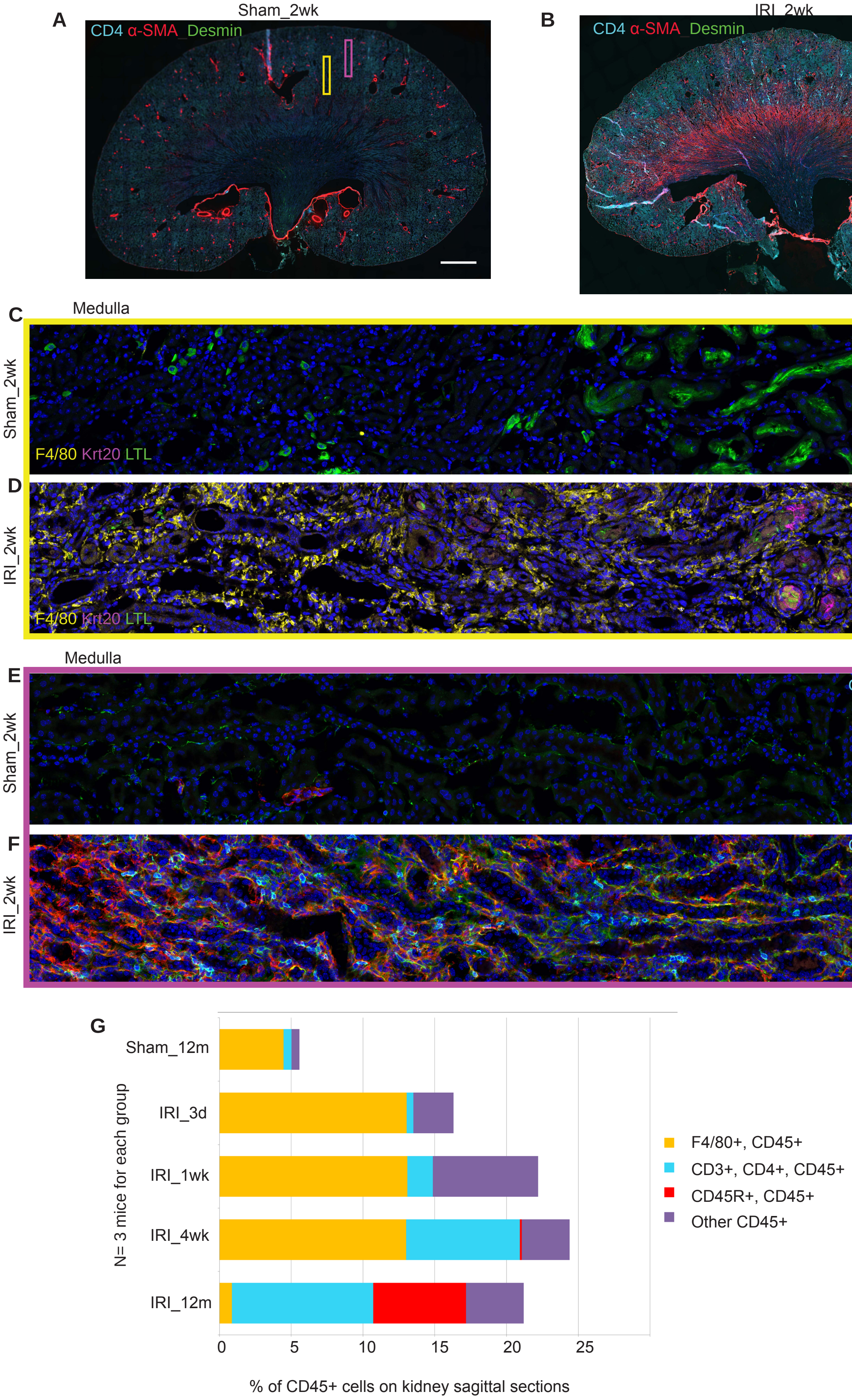

Supplemental Figure 6: Expression pattern of representative down-regulated gene *Mfsd2a* post IRI from module VII. Whole kidney view of sagittal sections of kidneys 2 weeks post sham(A) and IRI (B) through RNA section in situ. (C) Zoomed in view of RNA section in situ co-stained with LTL in the outer stripe of outer medulla of sham kidney. Split channels (D-F) and merged image (G) were from of the red square region in (C). Scale bar 1mm (A-B), 200µm (C), 50µm (D-G).

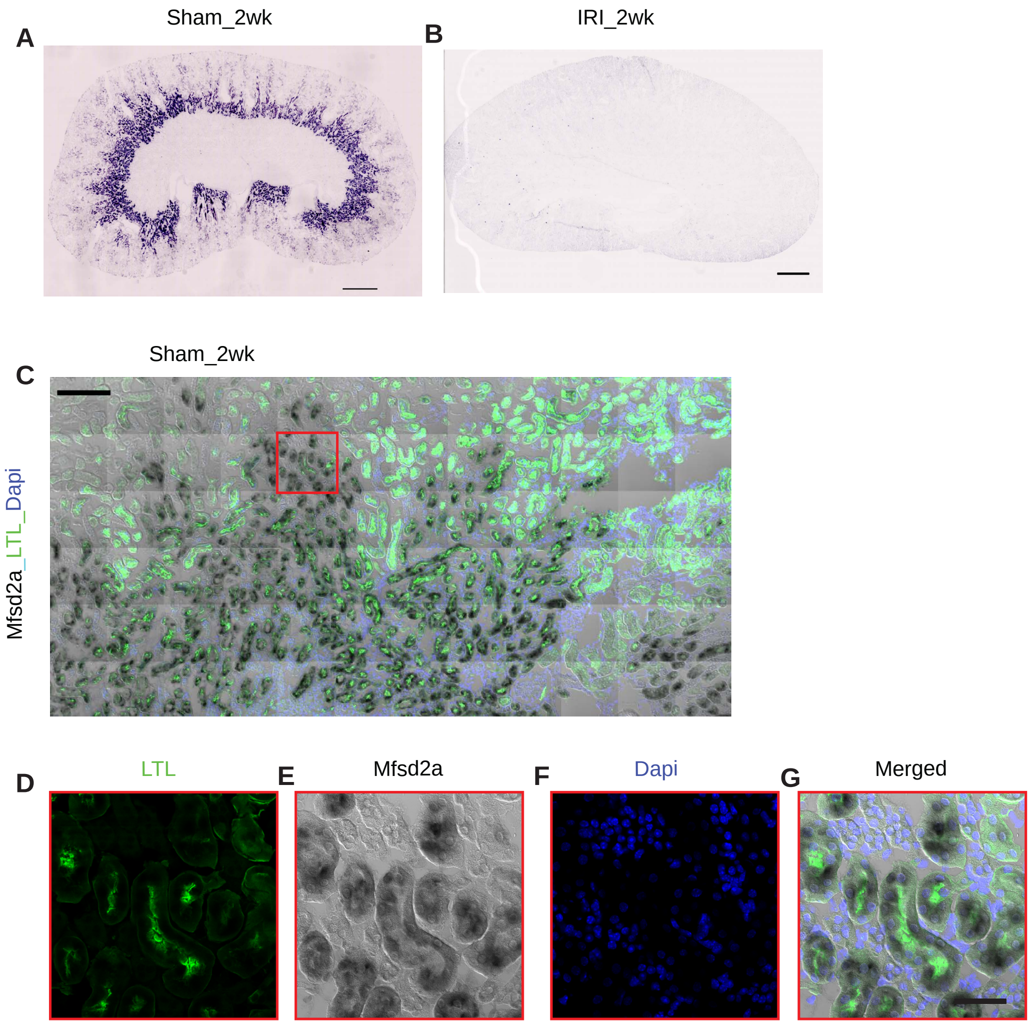

Supplemental Figure 7: RNA-seq sample clustering (A) and heatmap of expression profiles in mouse renal IRI samples for differentially expressed genes identified in published human liver transplant 2 to 4 hours post reperfusion. Left columns indicated relative expression in human samples. Detail sample information for individual column is the same as that in Figure 3B.

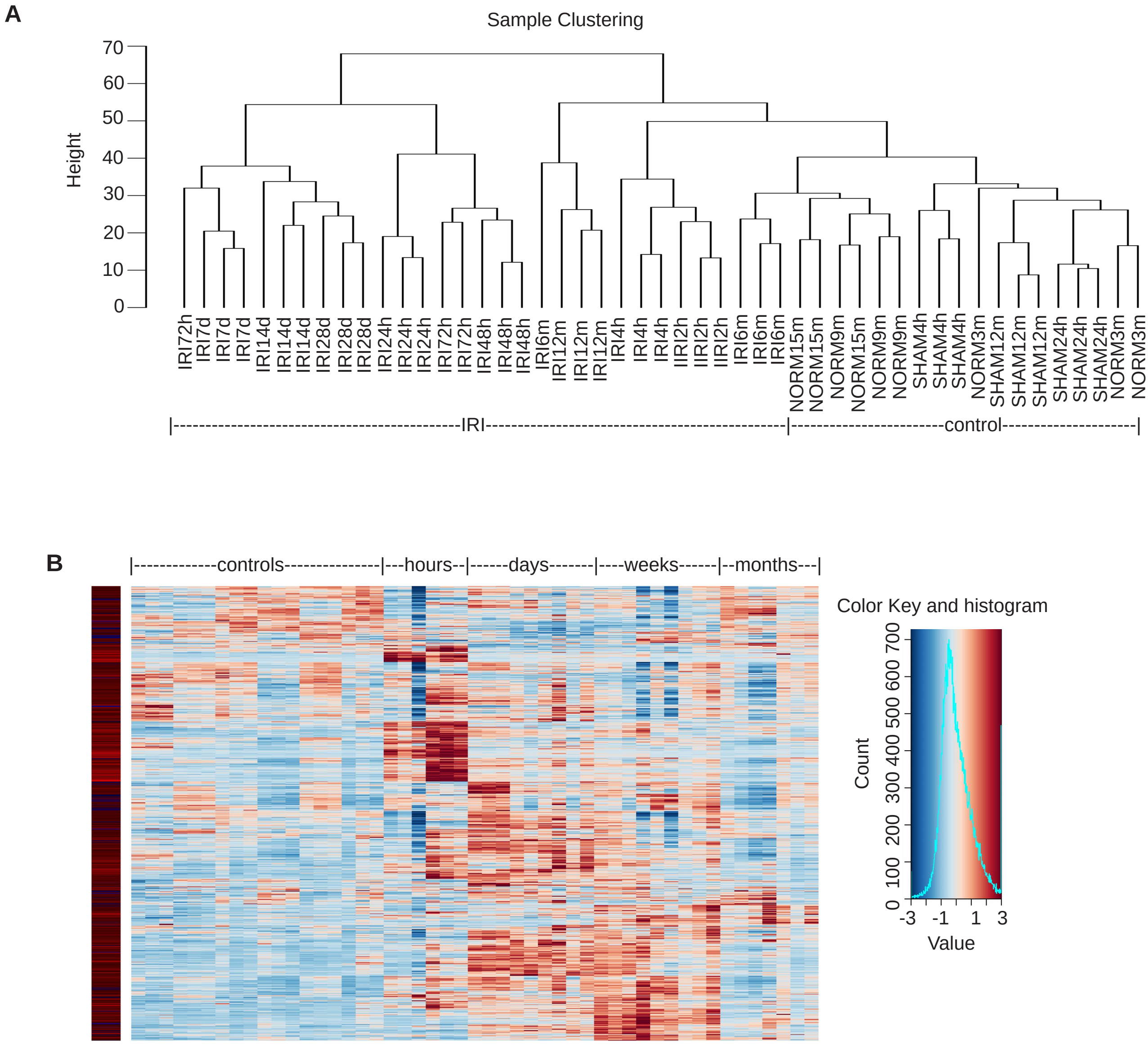

Supplement: Supplemental figures [file jciinsight-2-94716-s002.pdf]
